# An Introduction to Smart Home Ward–Based Hospital-at-Home Care in China

**DOI:** 10.2196/44422

**Published:** 2024-01-30

**Authors:** Weibin Cheng, Xiaowen Cao, Wanmin Lian, Junzhang Tian

**Affiliations:** 1 Institute for Healthcare Artificial Intelligence Application, Guangdong Second Provincial General Hospital, Guangzhou, China; 2 Information Department, Guangdong Second Provincial General Hospital, Guangzhou, China

**Keywords:** smart home ward, telemonitoring, telemedicine, home care, hospital at home, healthcare delivery, implementation, smart ward, medical monitoring, medical care, rehabilitation, health care

## Abstract

Hospital-at-home has been gaining increased attention as a potential remedy for the current shortcomings of our health care system, allowing for essential health services to be provided to patients in the comfort of their own homes. The advent of digital technology has revolutionized the way we provide medical and health care, leading to the emergence of a “hospital without walls.” The rapid adoption of novel digital health care technologies is revolutionizing remote health care provision, effectively dismantling the conventional boundary separating hospitals from the comfort of patients’ homes. The Guangdong Second Provincial General Hospital has developed a 5G-powered Smart Home Ward (SHW) that extends medical care services to the home setting and is tailored to meet the needs and settings of each patient’s household. The SHW was initially tested for its suitability for treating 4 specialized diseases, including cardiovascular disease, stroke, Parkinson disease, and Alzheimer disease. Understanding and addressing the potential challenges and risks associated with SHWs is essential for the successful implementation and maintenance of safe and effective home hospitalization.

## The Future of Older Adult Care in China: Innovations in Health Care Delivery

China has emerged as a rapidly aging society. Aging contributes significantly to the health care burden in China due to the increased prevalence of chronic diseases and disabilities among older adults. The health care burden for the population of older individuals in China is further compounded by inadequate health care infrastructure and limited access to health care services in rural areas. The Chinese government has implemented various policies and initiatives to address these challenges, including expanding health care coverage, promoting preventative care, and increasing investment in health care infrastructure. Currently, the top-level design of China’s older adult care model is “9073,” which means that 90% of older people receive older adult care services at home, 7% of them receive short-term care in the community, and 3% of them receive institutional care. This implies that the age-friendly and livable environment at home affects 90% of the population of older adults.

Hospital-at-home, which seeks to provide essential health services to patients in the comfort of their own homes, has been gaining increasing attention as a feasible solution for at-home older adult care and medical services [1-3]. The COVID-19 pandemic has underscored the risks of overreliance on physical medical institutions, emphasizing the continued need to develop a decentralized medical service ecosystem that revolves around patients’ families and communities. Hospital-at-home programs have been implemented in many high-income countries for years [4-7]; however, successful programs are limited [8]. Experience translating from high-income countries to low- and middle-income countries can be challenging, where medical resources are limited and public health literacy is low [2,3,9,10].

## The Growth of Emerging Technologies for Home Hospitalization

New digital health care technologies are being rapidly adopted for remote health care provision, which is breaking down the traditional barrier between the hospital and home. The emergence of 5G technology posed the potential to further enhance home hospitalization by enabling remote patient monitoring and real-time communication between health care providers and patients. The introduction of sensor-based wearables and devices has changed the way that clinical data are collected and stored, leading to groundbreaking advancements in how care is provided [11,12]. Digital biomarkers—a set of objectives, quantifiable measures of physiological and behavioral characteristics that are acquired via wearables, implants, and other devices—are becoming increasingly essential in this process [13,14]. Home hospitalization can greatly benefit from the use of digital biomarkers, providing care teams with a more comprehensive understanding of a patient’s health. These biomarkers enable the tracking of vital signs such as heart rate and respiration rate, as well as changes in sleep patterns, activity levels, and dietary habits [12,15,16].

## Smart Home Ward for Hospital-at-Home Care

**Figure 1.**
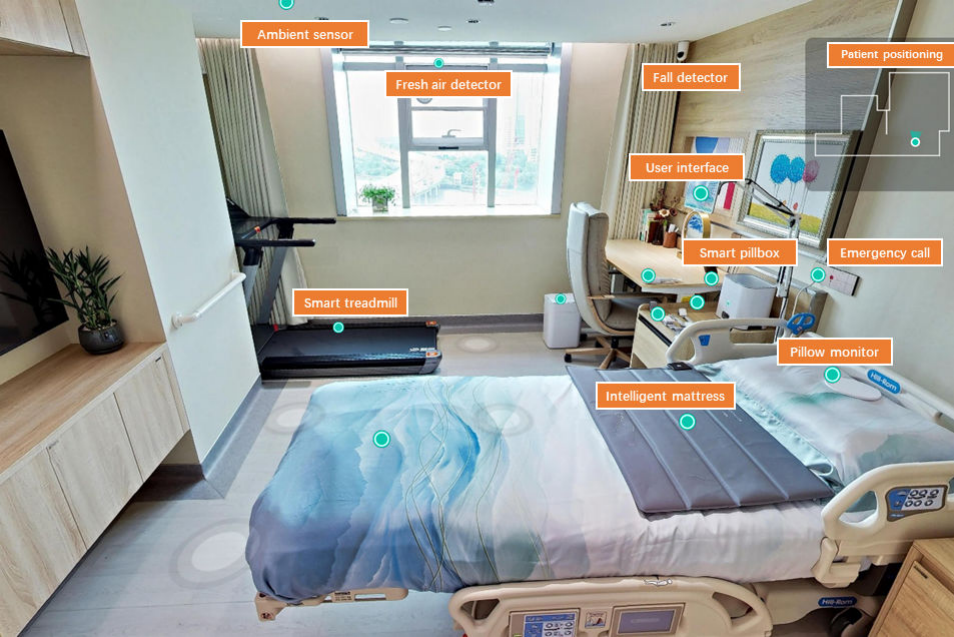
Demonstration of the Smart Home Ward in the hospital.

In 2021, Guangdong Second Provincial General Hospital developed the “Hospital Intelligent Twins,” a 5G-powered smart hospital that integrates the Internet of Things (IoT), artificial intelligence (AI), cloud computing, and 5G applications to create all-scenario intelligence for health care and hospital management [17,18]. Leveraging this 5G smart hospital infrastructure, Guangdong Second Provincial General Hospital further explored the Smart Home Ward (SHW), which seeks to break through the “wall” of centralized hospital-based health care services by extending care services to the home setting (Figure 1). The SHW is a seamless hospital unit managed by health professionals in the hospital and is designed to provide patients with equivalent hospital-level services at home, including medical monitoring, ward rounds, consultation, and medical care, and so on. SHW can support 2 hospital-at-home models, early-supported discharge and admission avoidance, offering integrated health monitoring, medical care, and rehabilitation services all within the comfort of their homes [19,20].

The management of patients in the SHW is handled by an interdisciplinary care team overseen by responsible specialists from relevant clinical departments. At present, the Departments of Cardiology and Neurology are pilot testing SHWs for patients with cardiovascular conditions and those with cerebrovascular conditions, respectively. Each specialty establishes a dedicated home ward team comprising physicians from that department, along with nurses, pharmacists, physical therapists, occupational therapists, and social workers. For example, the post–coronary heart disease treatment team includes cardiologists, cardiac nurses, and rehabilitation therapists. Patients undergo an initial clinical assessment by their specialist physician to determine their suitability for remote home care. The inclusion and exclusion criteria for specific conditions such as coronary heart disease and stroke are provided in Multimedia Appendix 1. If deemed feasible based on the assessment and if the patient provides consent, the interprofessional SHW staff conduct a home environment evaluation. Necessary modifications are made and monitoring equipment or devices installed to safely support care at home with remote specialist oversight.

In the SHW, series of digital biomarkers are monitored, including blood pressure, heart rate, respiratory rate, blood oxygen saturation, body temperature, and electrocardiographic signals, with patients’ daily activities, treatment compliance, and risk occurrences monitored via contact or contactless devices. These data can be leveraged to tailor treatments or notify care providers of any deviations from expected parameters.

Data generated from the SHW are transmitted securely to the hospital’s electronic medical record (EMR) system in real time. The integration follows privacy and security guidelines set forth by our hospital’s Health Insurance Portability and Accountability Act (HIPAA)–compliant policies. Only deidentified data points that are relevant for clinical care, such as vital signs, activity levels, and medication adherence data, are integrated into the EMR. Data transmission from the SHW to the EMR is one-way to ensure the security of sensitive hospital information. Authorized care team members, including attending physicians, nurses, pharmacists, physical therapists, and case managers, have access to consolidated patient data reports within the EMR system. This allows them to monitor trends, recognize any deviations from normal ranges, and act accordingly without needing to use separate systems.

The primary care team, led by the patient’s attending physician, is responsible for reviewing the daily reports and contacting patients if follow-up care is needed, based on the remote patient monitoring data. Family members and caregivers participate in care conferences to stay updated on the patient’s progress but do not have access to the EMR. We aim to expand access to aggregated reports to allow for greater caregiver involvement while maintaining privacy and security.

A pretraining ward has been established in the hospital to help patients transit from hospital to home living. Patients are trained on the use of smart home devices and receive guidance on remote rehabilitation training. In addition, the pretraining ward includes medical assessments to ensure that the patient is eligible for admission to SHW care and will benefit from the treatments provided at home. Specialized staff consisting of nurses, physical therapists, and occupational therapists are dedicated to supporting the SHW program. These clinicians provide personalized patient education, demonstrations, and skills training. They ensure that patients and caregivers are comfortable with the remote monitoring system and therapy program prior to discharge. The goal is to maximize treatment adherence and outcomes through empowering patients and families with knowledge. A dedicated tech support team is also available to assist families in successfully setting up the in-home system and addressing any technical issues that may arise post discharge. Readily available support from clinical and technical experts further enables safe and independent living at home with remote care and monitoring.

## Characteristics of the SHW

The SHW constructed in this project not only integrates advanced medical IoT technology and products, but also involves the renovation of the environment to make it suitable for older adults and patients, extending homogeneous medical services provided in the hospital wards to patients’ homes. Its characteristics are mainly reflected in the following 4 aspects.

First, the SHW has a multitude of functions including ambient sensing, medical monitoring, rehabilitation training, exercise and diet guidance, psychological counseling, and sleep management. To achieve these capabilities, a variety of cutting-edge technologies such as 5G or Wi-Fi 6, Internet of Medical Things (IoMT), smart wearable devices, smart home appliances, and health monitoring equipment have been deployed in the home setting. For instance, an intelligent closetool can detect urine levels and monitor heart rate, body fat, and the length of time spent on the toilet [21]. Additionally, an intelligent mattress and pillow monitor heart rate, respiration rate, and body movement frequency during sleep [22,23]. Applications such as those for fall detection in the bathroom and the amount of stillness in daily activities can trigger an automatic alarm for an emergency, which connects to the hospital [24].

**Figure 2.**
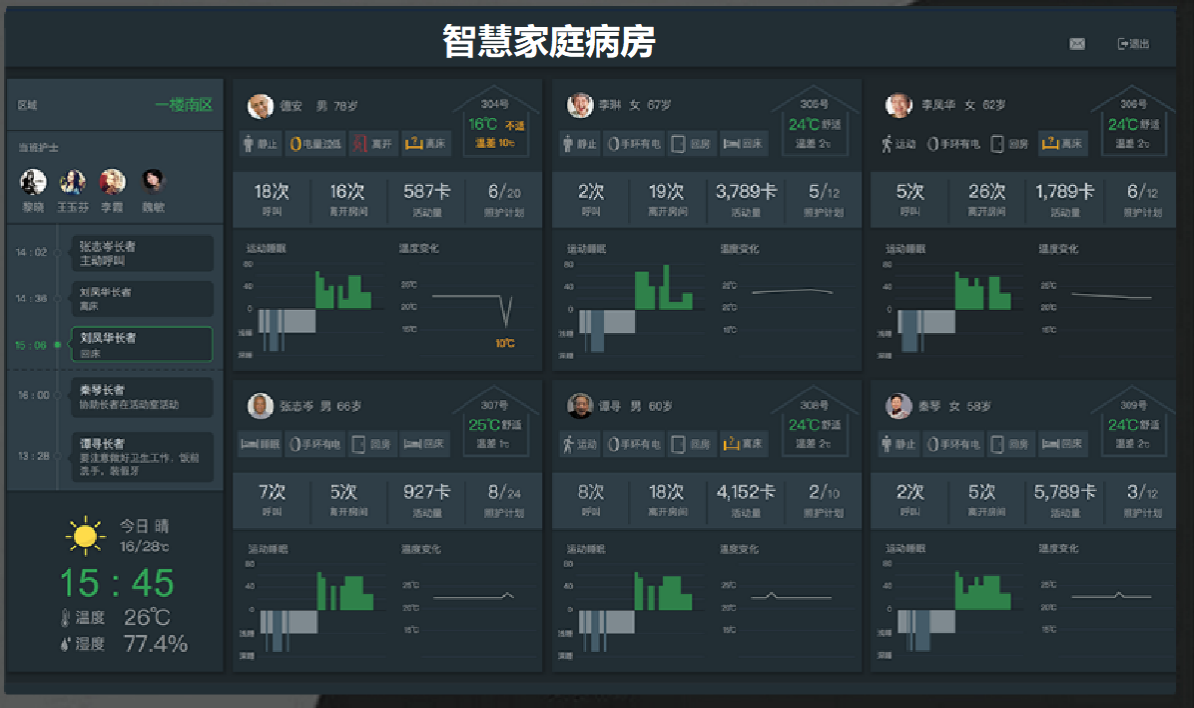
Screenshot of the Smart Home Ward Medical Management Information System.

Second, to facilitate hospital-at-home management, a platform was developed with the integration of cross-system and cross-ecological IoMT devices (Figure 2; see the screenshot of the SHW Medical Management Information System). Thus, IoMT devices and ambient sensors are interconnected, and data are generated, gathered, managed, and processed by various terminals, including data from a hospital and a SHW (including data for the home environment, daily activities, treatment, rehabilitation, etc). This enables interoperability for home ward management, allowing patients, health professionals, and caregivers to use mobile terminals such as tablets to view the patient’s physiological data in real time and manage the home ward setting with ease. As medical teams have the capability to monitor a patient’s health from a distance, fewer in-person visits are needed, thus decreasing the cost of care [25].

Third, deployment of an SHW is tailored to cater the needs and settings of a patient’s household. To promote patient autonomy in the home setting, the SHW undergoes various levels of renovations suitable for aging individuals, creating a barrier-free, safe, and secure living space. Furthermore, ambient sensors enable automatic configuration to ensure the comfort of the environment [26]. These sensors continuously detect the air, temperature, and humidity in the ward. Whenever the indoor concentrations of particulate matter of diameter ≤ 2.5 μm, formaldehyde, and carbon dioxide exceed standard levels, the SHW automatically adjusts its equipment to improve indoor air quality.

Fourth, the extension of hospital care services to the home setting is achieved through the integration of various technologies and resources. This challenge is addressed by the SHW through the deployment of advanced IoMT technology and products, which allow for the passive monitoring of patients’ health and the implementation of timely medical interventions. Additionally, a comprehensive EMR system has been implemented, enabling seamless sharing of patients’ medical records between the hospital and home health care systems. Furthermore, a robust 5G-based telemedicine infrastructure has been established, enabling real-time communication between patients and doctors, including digital consultations, remote monitoring, and telemedicine-enabled home visits, as well as providing on-demand home care services [27].

## SHW Care Plans for Specialized Diseases

The SHW’s feasibility of treating 4 specialized diseases, namely Parkinson disease, cardiovascular disease, stroke, and Alzheimer disease, is being tested. For each disease, corresponding care plans have been developed, and necessary monitoring and treatment facilities have been suggested.

In the case of Parkinson disease, exercise rehabilitation treatment is mainly used as the primary treatment modality in the home ward. Patients are guided by doctors remotely to implement various forms of internet-based rehabilitation exercise training, such as relaxation training, joint range of motion training, muscle training, breathing training, gait training, balance training, and cognitive training. During exercise, the patient’s heart rate is monitored using a smart watch to prevent excessive exercise intensity. A smart lunch box is provided for daily monitoring of the frequency and amplitude of the patient’s hand tremors while eating to evaluate the effectiveness of rehabilitation treatment [28].

For patients with cardiovascular disease, the home ward caters to those with ST-segment elevation myocardial infarction, non–ST-segment elevation acute coronary syndrome, stable angina pectoris, ischemic cardiomyopathy, chronic systolic heart failure, and sudden cardiac death syndrome. Cardiac rehabilitation exercise therapy is guided by health professionals from the hospital, which includes aerobic exercise, resistance training, and neuromuscular training. A treadmill with heart and lung function monitoring is set up as a rehabilitation tool at home, which records and monitors the patient’s exercise status and heart rate, blood oxygen saturation, and calorie consumption. This allows doctors to adjust the program promptly [29].

For stroke, given that individuals who have experienced a stroke often face difficulties with mobility, the adaptation of living spaces to suit their needs is crucial to promote independent living and facilitate rehabilitation. To this end, we have implemented appropriate accessibility retrofits within the home setting to establish a barrier-free environment for patients. Such efforts can contribute to not only increased mobility but also heightened patient confidence. To evaluate the efficacy of functional rehabilitation, regular assessments are conducted through both home visits and hospital evaluations.

In the case of Alzheimer disease, a comprehensive treatment approach is adopted, including cognitive training, task training, and music therapy. These treatments are supported by the augmented reality and virtual reality applications that allow doctors to simulate complex scenarios to stimulate patients’ brain activities in a safe and controlled environment. Several physiological signals, such as heart rate variability, eye movements, and sleep patterns, are recorded and are used to train models for treatment evaluation. Furthermore, an AI-powered camera is installed in the home environment to monitor the patient’s activity status, such as sitting and lying time. A smart watch with GPS positioning and communication functions is also used to prevent patients from getting lost [30].

## Challenges for the Scale-Up of the SHW Care Program in China

The widespread adoption of SHWs has been faced with several challenges. First, authoritative guidelines for the implementation of medical service standards for home ward care and those for setting up technology-enabled digital wards in China are lacking. Second, a sustainable fee-based model for home ward care has yet to be established. Third, the effective operation of home ward care requires close collaboration among hospitals, community health service organizations, and family members. However, there are currently no unified regulations to delineate the responsibilities of various stakeholders involved in providing home ward care. Fourth, the popularity and reliability of the technology needs improvement. SHWs require specialized equipment and technologies, a secure and trust-based environment, and staffing competencies to ensure patient safety and privacy. A unique standard needs to be established to ensure the interconnectedness and interoperability of various devices. Meanwhile, potential problems associated with the cost of equipment, access to necessary data, and data privacy also exist.

## Implications of Hospital-at-Home Care Using SHWs for Practitioners, Researchers, and Policy Makers

The implementation of SHWs has the potential to extend the quantity and quality of hospital care services in response to the increasing demand for medical care in China’s aging society. For health care practitioners, the integration of smart home digital health technologies can facilitate remote patient monitoring and management, enabling timely and effective care delivery in patients’ homes. This approach can reduce the burden on hospitals and clinics, while improving patient outcomes and satisfaction. To enable the successful implementation of SHWs, new team structures with corresponding workflows must be created in clinical settings to optimize health care systems and patients’ usage of this technology. Clinical physicians must work closely with technicians in developing workflows and integrated AI tools and in the process of care provision, such as remote monitoring.

For researchers, the implementation of SHWs presents a unique opportunity to study the impact of digital health technology on health care delivery, patient outcomes, and cost-effectiveness. However, ethical considerations must be navigated when accessing the vast amounts of data gathered at SHWs to ensure patient privacy and security [31]. All stakeholders must understand and adapt to these implications to fully harness their potential benefits while mitigating associated risks.

Policy makers can benefit from the use of SHW technologies, as they show promising potential in reducing health care costs and improving access to care, particularly for patients in rural or underserved areas. However, policy makers must establish regulations to safeguard patient privacy and security, ensure equitable access to technology, and enhance digital infrastructure for widespread adoption. With careful consideration of these factors, the implementation of SHW can revolutionize hospital-at-home care, improving the quality of care, reducing health care costs, and ultimately benefiting patients and the health care system as a whole.

## In Summary

The advent of digital technology has revolutionized the way we provide medical and health care, leading to the emergence of “hospitals without walls.” This SHW is an innovative care model that promises to bring a host of improvements to health care by providing convenient access to “boundless ambulatory care,” as well as “boundless inpatient care.” Despite the potential benefits, these digital projects are still faced with some challenges, such as public acceptance and adoption of the technology, willingness to pay for services, and encouraging medical insurance uptake. Therefore, it is essential for governments to promote public awareness of the advantages of digital technology and introduce incentives that motivate people to take up medical insurance in order for these services to be widely available.

## Supplementary material

10.2196/44422 Multimedia Appendix 1 Inclusion/exclusion criteria for specific conditions.

## Abbreviations

AI: artificial intelligence
EMR: electronic medical record
HIPAA: Health Insurance Portability and Accountability Act
IoMT: Internet of Medical Things
IoT: Internet of Things
SHW: Smart Home Ward
