# Supplementary material for: An Introduction to Smart Home Ward–Based Hospital-at-Home Care in China
Source: JMIR Mhealth Uhealth. 2024 Jan 30;12:e44422. doi: 10.2196/44422 (PMC10850850; doi:10.2196/44422)
Supplement: Multimedia Appendix 1 [file mhealth-v12-e44422-s001.docx]

**Supplementary materials**

【Recruitment Criteria for Coronary Heart Disease Patients】

Coronary heart disease is a type of heart disease caused by stenosis or occlusion of the coronary arteries. In addition to drug and surgical treatments, home medical monitoring, rehabilitation training, exercise guidance, dietary adjustment, psychological counseling and sleep management are also very important for patients.

Potential participants for the smart home ward project for coronary heart disease patients must meet the following criteria:

1. Age between 40-70 years old, weight between 40-80kg, gender is not limited,
2. Permanent residency in Guangzhou city;
3. Definitive diagnosis of "acute ST-segment elevation myocardial infarction" with time from onset ≤12h, and received emergency percutaneous coronary intervention treatment;
4. Time from initial diagnosis of acute myocardial infarction <1 month, and received standard secondary prevention pharmacotherapy for coronary heart disease continuously since onset;
5. No cardiopulmonary resuscitation received within the past 1 month, without severe ischemic or hemorrhagic complications, severe infections, pulmonary embolism, severe liver dysfunction, intracranial lesions and/or neurological or psychiatric diseases, malignant tumors, or autoimmune related diseases;
6. Functioning normally in daily activities, echocardiogram suggests preserved left ventricular systolic function with 45%≤EF≤55%;
7. A certain level of education and cognitive ability that can communicate with the physician;
8. Good self-management skills;
9. Availability of a stable caregiver;
10. Voluntary participation, and signed informed consent and relevant legal documents by their guardian.

【Recruitment Criteria for Stroke Patients】

Stroke is a common disease with high rates of disability and mortality. In addition to drug and surgical treatments, home medical monitoring, rehabilitation training, exercise guidance, dietary adjustment, psychological counseling and sleep management are also very important for patients.

Potential participants for the smart home ward project for stroke patients must meet the following criteria:

1. Stroke patients diagnosed by CT or MRI within 90 days, meeting the diagnostic criteria of 《Diagnostic Criteria for Major Cerebrovascular Diseases in China 2019》, including cerebral infarction (excluding lacunar infarction and cerebral infarction), intracerebral hemorrhage (excluding cerebral microhemorrhage and cerebellar hemorrhage), mixed cerebrovascular diseases;

2. Aged between 30-75 years old, gender is not limited;

3. Stroke patients 15-90 days after onset with clear neurological dysfunction manifestations of moderate stroke, such as motor dysfunction (National Institutes of Health Stroke Scale score of 5);

4. Stable condition and mood during rehabilitation;

5. Can communicate normally without cognitive deficits and able to follow research procedures.

The following types of patients will not be included in the recruitment:

1. Those with implanted cardiac pacemakers, heart valve replacements or intracranial stents, intracranial aneurysm clips, or deep brain electrodes etc.;

2. Those with a history of epilepsy;

3. Those with severe heart, liver, kidney or lung disease;

4. Those with other diseases affecting gait and balance such as musculoskeletal disorders, lower limb vascular diseases, neurological/muscular diseases or traumatic diseases;

5. Cases where patients or their families cannot provide sufficient cooperation during rehabilitation training;

6. Those with unstable mood while taking psychiatric medications;

7. Alcoholics;

8. Pregnant or lactating patients.
